# Omega Fatty Acids and Inflammatory Bowel Diseases: An Overview

**DOI:** 10.3390/ijms20194851

**Published:** 2019-09-30

**Authors:** Ledyane Taynara Marton, Ricardo de Alvares Goulart, Antonelly Cassio Alves de Carvalho, Sandra Maria Barbalho

**Affiliations:** 1 Department of Biochemistry and Pharmacology-Medicine, School of Medicine, University of Marília, Av. Higino Muzzi Filho 1001, Marília 15525-902 São Paulo, Brazil; ledyanemarton@hotmail.com; 2 Gastroenterology Department, University Hospital- Associação Beneficente Hospital Universitário -UNIMAR-Marília, 15525-902 São Paulo, Brazil; ricardogoulartmed@hotmail.com; 3 Postgraduate Program in Structural and Functional Interactions in Rehabilitation-UNIMAR-Marília, 15525-902 São Paulo, Brazil; silmaraebarbalho@gmail.com; 4 Food Technology School, Marília 17500-000 São Paulo, Brazil

**Keywords:** omega 3, eicosapentaenoic acid, docosahexaenoic acid, ulcerative colitis, crohn’s disease

## Abstract

Inflammatory bowel diseases (IBD) are chronic, inflammatory processes that affect the gastrointestinal tract and are mainly represented by ulcerative colitis (UC) and Crohn’s disease (CD). Omega 3 (ω3) fatty acids (eicosapentanoic acid and docosahexaenoic acid) show an indispensable role in the inflammatory processes and, for these reasons, we aimed to review the effects of these acids on UC and CD. Databases such as PUMED and EMBASE were searched, and the final selection included fifteen studies that fulfilled the inclusion criteria. The results showed that ω3 fatty acids reduce intestinal inflammation, induce and maintain clinical remission in UC patients, and are related with the reduction of proinflammatory cytokines, decrease disease activity and increase the quality of life of CD patients. Furthermore, the consumption of these fatty acids may be related to a reduced risk of developing IBD. Many studies have shown the beneficial effects of ω3 as adjunctive in the treatment or prevention of UC or CD. Nevertheless, most were performed with a small number of patients and there are many variations in the mode of consumption, the type of food or the type of formulation used. All these factors substantially interfere with the results and do not allow reliable comparisons.

## 1. Introduction

The immune system plays an indispensable role against infectious and inflammatory processes by reducing and extinguishing the stimuli or removing the damage to the tissues. Many disorders are associated with uncontrolled inflammation, such as rheumatoid arthritis, cardiovascular disorders, cancer, and inflammatory bowel diseases (IBDs) [1,2]. 

IBDs are debilitating, chronic, relapsing, and remitting inflammatory processes that affect the gastrointestinal tract and are mainly represented by ulcerative colitis (UC) and Crohn’s disease (CD). More than 3 million people are estimated to be affected in the United States, 2.5 million in Europe, and 75,000 in Australia [3], which incurs a relevant burden to the public health systems. 

These inflammatory conditions occur due to an imbalance in the intestinal immune response to intestinal microbes or other environmental conditions, resulting in a disturbance between pro- and anti-inflammatory molecules, as well as several other factors that may be involved in the chronic inflammatory state. These factors include cytokines, interleukins (ILs), activated toll-like receptors (TLR), nitric oxide (NO), free radicals, oxylipins, and the intestinal microbiota itself [4,5,6,7]. 

The synthesis and release of these mediators result in an onset of triggering factors and the beginning of repairing the injured tissues. These processes are followed by chemotaxis and recruitment of polymorphonuclear neutrophils and monocytes that start and maintain the inflammatory tissue reaction. At this stage, the production of specialized pro-resolving mediators derived from omega-3 (ω3) polyunsaturated fatty acids (FA) is also observed, which are useful in the resolution of inflammation. Several studies have shown that these pro-resolving mediators may become valuable tools for the understanding and treatment of IBDs [1,8]. 

ω3 fatty acids are essential to human nutrition. However, the Western diet is characterized by an imbalance between the intake of ω3 and ω6 (these acids are described below in the Section 3.2). The consumption of linoleic acid (LA, ω6) increased around three times over the 20th century. Numerous epidemiological studies have highlighted the role of dietary intake of monounsaturated fatty acids (MUFA) or polyunsaturated FA (PUFA) in the development of UC and CD. Higher intake of LA is associated with an increased risk of both diseases, whereas consumption of cocosahexaenoic acid (DHA) and eicosapentaenoic acid (EPA) is considered beneficial. In animal models, the use of ω3 regulates the peroxisome proliferator-activated receptor/nuclear factor of activated T cells (PPAR-γ/NFAT) and aids in the healing of intestinal mucosa [9,10,11,12,13,14,15]. 

The complex multifactorial etiopathogenesis of IBDs, alongside their relapsing and remitting nature, make them challenging to treat. Currently, the standard treatment mainly includes the use of corticosteroids, immunosuppressants, antibiotics, and biological agents that make the cost of this treatment expensive worldwide. On the other hand, these medications do not always lead to remission and may be related to numerous side effects. [16]. Thus, the use of therapies that can assist in the treatment and improvement of the quality of life of the patient is necessary. For these reasons, we aimed to review the effects of ω fatty acids on UC and CD.

## 2. Results

Table 1 shows fifteen studies [4,17,18,19,20,21,22,23,24,25,26,27,28,29,30] with the final selection of the survey: one randomized clinical trial, four randomized controlled clinical trials, five case-controls, one pilot study, two clinical trials, and two prospective cohort studies. These studies included 1,189 patients; 334 with CD, 488 with UC, and 367 in controls. From these patients, 568 were women (136 with CD; 242 with UC, and 190 controls) and 621 were men (198 with CD, 246 with UC, and 177 controls). The age range in UC patients was from 14 to 70 years, in CD patients from 5–61 years, and in controls from 10 to 86 years. Besides that, two prospective studies were included in the table, Chan et al. [22] and Ananthakrishnan et al. [23]. These two studies involved 3,317,845 people, among them 411 developed UC and 269 developed DC (age range was 20–74 years).

## 3. Discussion

### 3.1. IBD: Pathophysiologic Aspects

The relationship between the immune system, microbiome, genetics, and environmental factors is just beginning to be enlightened. Literature shows that there are over 200 IBD susceptibility genes [31,32]. 

The increase of the intake of sugar, fats, and additives; reduction in the fiber content; insufficiency of vitamins such as A and D; and presence of oxidative stress are possibly the main factors that link lifestyle with UC and CD. These pathologies share similarities and differences in histological patterns and the release of cytokines. UC patients usually exhibit stratified patterns of inflammation, affecting areas from colon to rectum and limited to mucosal layer. CD patients show skipped areas of transmural inflammation that may affect mouth to anus. The terminal ileum is affected typically in CD. Furthermore, clinical presentation of CD includes diarrhea, abdominal pain, bleeding, fever, weight loss, and there is a risk of complications such as stenosis, fistulae, and abscesses, while UC usually causes rectal bleeding, abdominal pain, fever, diarrhea, and weight loss [2].

**Figure 1:**
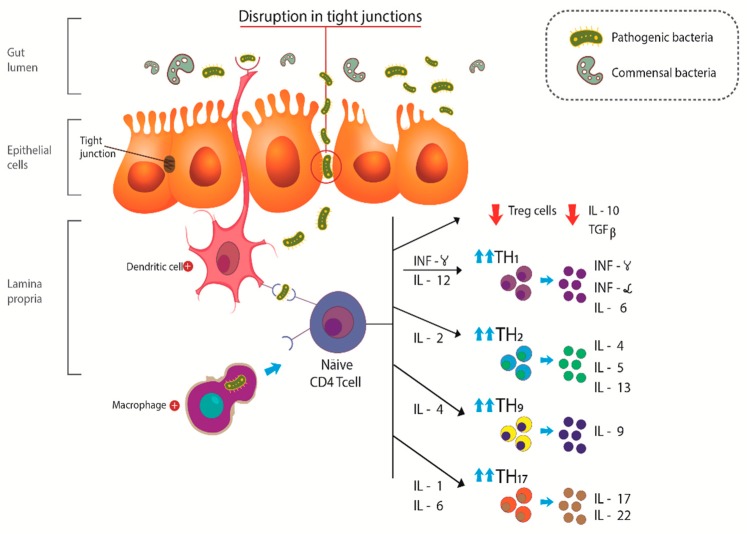
Pathophysiologic aspects of inflammatory bowel disease IBD. The disruption in tight junctions results in increased permeability of the epithelial cells leading to an augment in the uptake of antigens and activation of dendritic cells and macrophages, accompanied by decreased release of anti-inflammatory cytokines such as IL-10 and TGF-β, and increased release of proinflammatory cytokines such as INF-γ, TNF-α, IL-4, IL-5, IL-9, IL-17, and IL-22 (modified from Barbalho et al. 2018 [16]). IL: Interleukin; TGF-β: transforming growing factor-β; INF-γ: interferon-γ; TNF-α: tumor necrosis factor-α.

Both UC and CD show a disruption in the epithelial barrier, which results in an augmented intestinal permeability to commensal and pathogenic bacteria, leading to activation of TLR, dendritic cells, macrophages, and stimulation of the differentiation of näive T cells. These mechanisms of defense are triggered after the recognition of antigens by TLR and inappropriate activation of NFκB, resulting in activation of T helper cells (TH) such as TH1, TH2, TH9, and TH17. Activation of TH1 and TH17 occurs in CD and TH2 and TH9 in UC. Figure 1 exhibits the activation of TH cells and the release of pro-inflammatory mediators such as IL-4, IL-6, IL-9, IL-17, tumor necrosis factor-α (TNF-α), and interferon-γ (IFN-γ). On the other hand, a reduction in the release of anti-inflammatory IL-10 and transforming growing factor-β (TGF-β) was observed. The recently described IL-38 possess anti-inflammatory actions, inhibit TH17 maturation, and are associated with Treg activation. IBD patients seem to have abnormal expression of this interleukin, contributing to the inflammatory process. This scenario is associated with abdominal pain, bleeding, gas production, diarrhea, decrease quality of life, and leads to significant morbidity [33,34,35]. 

### 3.2. ω3 Fat Acids

Several authors have shown that dietary compounds may profoundly influence inflammatory processes in IBD patients. In addition to reducing fat and sugar intake and increasing fiber, fruit, and vitamin D content, much has been thought about the role of the polyunsaturated ω3 FA [36,37]. 

**Figure 2:**
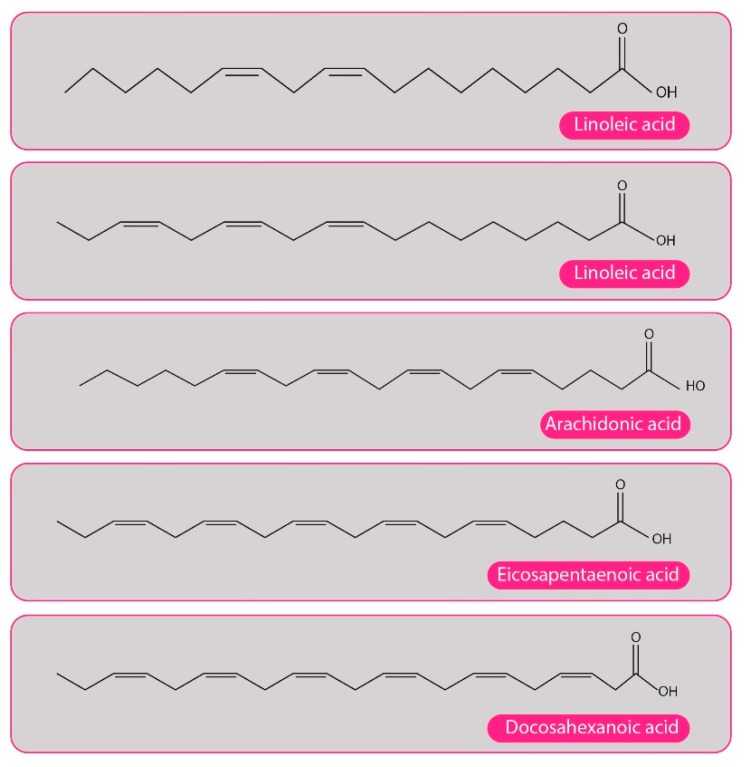
Structure of some fatty acids. ω6 (linoleic acid): first double bond at the sixty-carbon molecule from the methyl end of the chain; ω3 series (linolenic acid, C18:3; eicosapentaenoic acid, C20:5; docosahexaenoic acid, C22:6): first double bond at the third carbon molecule from the methyl end of the chain.

**Figure 3:**
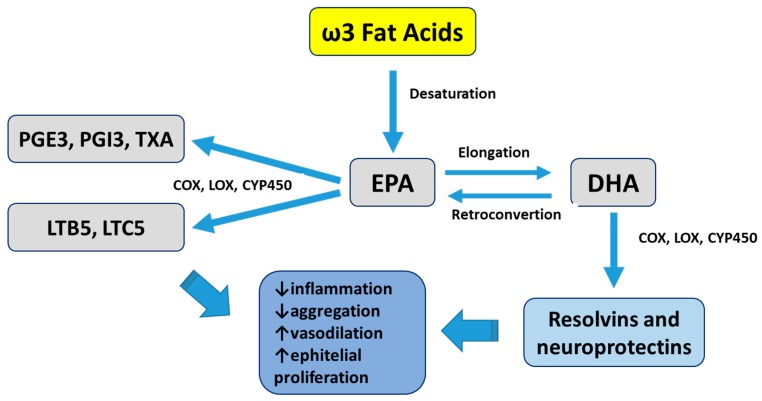
Eicosanoids from the ω3 family. ω3 series prostanoids are PGE3, PGI3, and TXA3; and ω5 series leukotrienes are LTB5 and LTC5. PGE3: prostaglandin E3; PGI3: prostaglandin I3; TXA3: thromboxane A3; LTB5: leukotriene B5; LTC5: leukotriene C5; COX: cyclooxygenase; LOX: lipoxygenase; CYP450: cytochrome P450 (modified from Barbalho et al., 2016 [2]).

The human body is capable of synthesizing all but two PUFAs: LA, that is precursor to the ω6 family, and α-linolenic acid (ALA), the precursor to the ω3 family compounds. The eicosanoids are produced from the ω6 and ω3 PUFAs and use ω6 arachidonic acid (AA) as the major substrate (Figure 2 shows the structure of some relevant fat acids). AA, LA, EPA, and DHA acids may lead to the production of substances known as oxylipins (resolvins, protectins, lipoxins, or maresins) (Figure 3) by several immune cells, such as macrophages. In addition to these mediators with anti-inflammatory and resolvin functions, there are also those with pro-inflammatory effects, such as prostaglandins and leukotrienes [1]. 

Oxylipins are produced through three major enzymatic pathways catalyzed by lipoxygenase (LOX), cyclooxygenase (COX), and cytochrome P450 (CYP450). LOX reactions produce leukotrienes (LT), hydroxyeicosatetraenoic acid (HETEs), and lipoxins (LX). COX produces prostaglandins (PG) and thromboxanes (TX), and CYP450 results in epoxyeicosatrienoic acids [38].

### 3.3. ω3 Fatty Acids and IBD

The role of oxylipins is far from being known in IBD, but many essential functions have been described, such as the ability to recruit neutrophils, potent chemotactic action (leukotriene B4: LTB4), platelet aggregation, increasing vascular permeability, and inducing epithelial proliferation after mucosal damage (prostaglandin E2 (PGE2), edema, and the release of pro-inflammatory cytokines such as TNFα, interleukin IL-1β, IL-6, and IL-8 [4,38,39].

Literature shows that the colonic mucosa of patients with active UC is linked to a significant increase in the availability of ω6, mainly AA, and decreased levels ω3, specifically EPA, and the ratio of AA/EPA [38,40]. 

Some clinical trials investigating the effects of fish-oil in IBD have shown beneficial results, such as a decrease of inflammation. On the other hand many of these studies fail to demonstrate effectiveness in preventing clinical relapse [41,42,43,44].

In a prospective multicenter cohort study, more than 200,000 individuals were screened for developing UC, and an amount of 126 individuals developed this condition. The higher intake of ω6 (LA) was associated with a doubling of the risk of UC in both genders. On the other hand, higher quantities of dietary ω3 (DHA) was associated with a lower risk of UC [45]. In the study by John et al. [46] with more than 25,600 participants, 22 UC cases were identified and negative association was found between increased intake of DHA and the risk of developing UC. The consumption of the LA was associated with an increased risk of UC. In a prospective cohort study with over 763,229 person-years of follow-up of 39,511 women, Ananthakrishnan et al. [11] found 103 incident cases of UC (14 new cases/100,000 per year) and 70 of CD (9 new cases/100,000 per year), and showed that diet may be associated with the risk of developing CD and UC. 

Table 1 includes other studies that investigated the associations of the PUFAS of the diet and risk of developing UC and CD, and studies showing the effects of the supplementation with FA in IBD patients. Below, we first present the studies related with UC, followed by those related to CD.

The investigation by Diab et al. [4] aimed to quantify bioactive metabolites of ω3 and ω6 polyunsaturated FA in the intestinal mucosa and in the cytokine gene expression during inflammatory events in UC. Authors highlighted the altered balance between pro- and anti-inflammatory lipid mediators in IBD and suggested potential targets for intervention using ω3-related substances. These results indicate the importance of PUFAs as alternative treatments for IBD. However, there is a need for investigations involving a larger sample, as the study was conducted with only 30 patients. Scaioli et al. [17] evaluated the ability of the EPA-free FA form (EPA-FFA) to reduce intestinal inflammation in UC patients and used fecal level of calprotectin as a marker. The study demonstrated that compounds derived from ω3 can, besides inducing, also maintain clinical remission for at least six months. Prossomariti et al. [18] showed that the supplementation with EPA-FFA reduced mucosal inflammation, promoted goblet cell differentiation, and modulated gut microbiota composition in UC patients. 

The study performed by Wiese et al. [20] showed differences in serum FA in UC individuals and controls, suggesting that these acids may affect cytokine production and thus be immunomodulatory in UC. However, subgroups for medication analysis were limited by the lack of treatment of näive patients, and therefore future large-scale experimental studies are needed to validate the assumptions made in the study. On the other hand, Grimstad et al. [29] showed that dietary PUFAs (obtained from Atlantic salmon), although promoting reduction in C Reactive Protein and homocysteine levels in UC patients, did not significantly change the levels of cytokines such as TNF-α. Further investigations would help to know the amount of Atlantic salmon that could be adequate to produce beneficial results. 

Another interesting study was performed by Pearl et al. [25]. These authors showed that modifications in the ratio of AA/EPA, in the amounts of AA, DPA, DHA, LA, α-LNA, and EPA are associated with the severity of inflammation in UC individuals. These findings are an open door to further interventions on supplementation with FA.

An open-label trial aiming to investigate the safety and efficacy of a ω3 emulsified formulation showed that it could be classified as safe with minimal side effects. CD activity index scores tended to decrease after ingestion of the formulation, and blood tests revealed no serious adverse effects [19]. Scaioli et al. [21] found that enteric-coated ω3 supplementation may be beneficial for UC patients and the use of EPA may work as a “universal donor” concerning the major ω3 PUFAs. Furthermore, such a formulation allows for long-term treatment and overcomes treatment with fish oil, which requires large cell dosage.

The susceptibility of developing CD may also be associated with variations of CYP4F3 and FADS2 genes, indicating that dietary and genetic interaction may influence disease pathogenesis. The study of Costea et al. [24] relates, for the first time, the relationship between these gene alterations, diet, and increased risk of developing CD. 

Bassaganya-Riera et al. [26] showed that the conjugated linoleic acid (CLA) supplementation was well tolerated and inhibited the ability of peripheral blood T cells to produce proinflammatory cytokines, decreased disease activity, and increased the quality of life of CD patients. 

Another study evaluated the use of a formulation with a higher concentration of LA, or higher concentration of ALA in pediatric CD patients, and showed that the group that received the first formulation showed 93% remission and the second group showed 79% [27]. Another formula including fish ω3 FA, prebiotics, and antioxidants led to significant decrease in AA phospholipid plasma levels with increased EPA and DHA levels, resulting in several benefits to CD patients [28]. These results indicate that the use of different formulations may be helpful in the therapeutic approach of CD patients.

Uchiyama et al. [30] also investigated the effects of diet therapy using a “PUFA ω3 food exchange table” (ω3DP) on the erythrocyte membrane FA composition of CD and UC patients and their remission-maintaining effects. ω3DP significantly increased the erythrocyte membrane ω3/ω6 ratio in IBD patients, and this ratio was considerably higher in the remission group, suggesting that ω3DP alters the composition of cell membrane FA and influences the clinical activity of IBD patients.

The cohort studies performed by Chan et al. [22] and Ananthakrishnan et al. [23] investigated the dietary intake of FA with validated food frequency questionnaires. The first study found positive associations between the consumption of DHA and prevention of CD. On the other hand, the second study, in general, showed that the intake of ω3 and ω6 did not influence the risk of developing UC or CD.

There are currently many studies showing the effects of FA as adjunctive in the treatment or prevention of UC or CD, but, except for cohort studies, most were performed with a small number of patients. In intervention studies, other biases that may be mentioned are the many variations in the mode of consumption, the type of food, or the type of formulation used. All these factors substantially interfere with the results and do not allow reliable comparisons.

Several studies have shown that diet is an essential factor in determining the gut microbiota composition. These findings suggest its fundamental role as an exogenous factor able to induce homeostasis or disruption of the bowel [47]. Since UC and CD remain incurable, the understanding of the role of ω3 in these inflammatory conditions may represent a new therapeutic target.

## 4. Methods

### 4.1. Data Sources

The authors of this review searched the MEDLINE-PubMed and EMBASE databases following the PRISMA guidelines (preferred reporting items for a systematic review and meta-analysis, Moher et al. [48]). This search was conducted to answer the following question: Is ω3 effective in treating or preventing Inflammatory Bowel Diseases?

### 4.2. Research

The research included randomized clinical trials, cohort studies, cross-sectional studies, case-control, and experimental studies. The combination of terms and keywords used for this search was “omega 3 and inflammatory bowel disease”, “alpha linoleic acid and inflammatory bowel disease”, “eicosapentaenoic acid and inflammatory bowel disease,” “docosahexaenoic acid and inflammatory bowel disease.”

**Figure 4:**
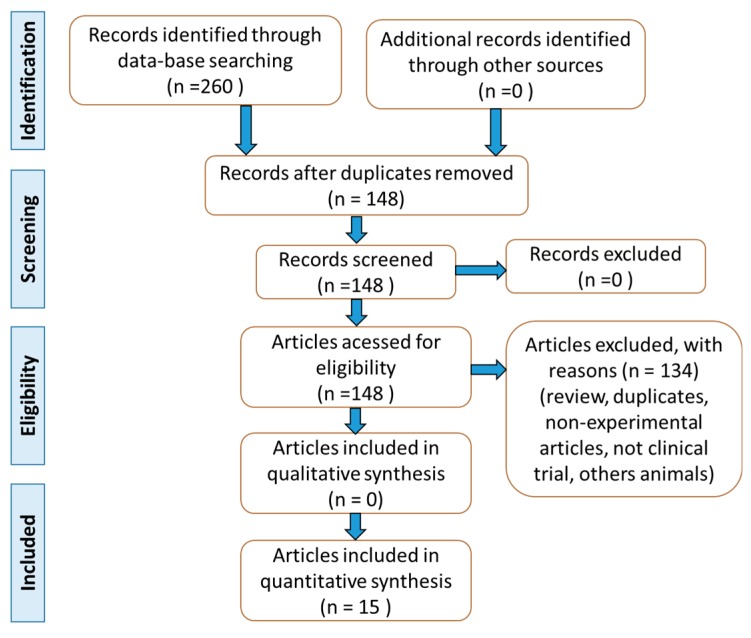
Flow diagram showing the results of the search according to PRISMA guidelines [48].

Based on the list of references obtained with the combination of these keywords, we built the flow diagram (Figure 4) that shows the selection of articles and inclusion and exclusion of studies. Other studies on Omega 3 and intestinal inflammatory disorders were used to build the discussion.

### 4.3. Eligible criteria and Study Selection

Our research included qualitative and quantitative studies that discuss the use of ω3 and its effects on the treatment of IBD. We have included English articles from the last ten years that showed correspondence with the keywords used for searching.

### 4.4. Extraction of Data

The extraction was performed independently by two authors who used the predefined inclusion and exclusion criteria, as well as the descriptors described above. Data were extracted from eligible articles that included: date, author, study design, sample size, gender, information related to the use of ω3, and its relationship with IBD. Only original articles were selected for the construction of Table 1. Inclusion criteria were articles that used randomized clinical trials, cohort studies, cross-sectional studies, case-control, and experimental studies. The exclusion criteria used for this search were non-English articles, case reports, poster presentations, and letters to the editor.

## 5. Conclusions

IBD is a condition associated with the quality of life of the patient and can be considered as a public health problem. Many studies have shown that ω3 FA are substrate to the production of protectins, resolvins, and maresins, which may regulate and attenuate the inflammatory processes and lead to remission of IBD and, thus, could be considered as a new complementary approach to the treatment of these inflammatory conditions.

However, there is still much controversy about the effects of these acids both on CD or UC, possibly due to the variability in the doses and way of delivery, in the size of the samples, and the biases found in different clinical trials. We suggest that further studies should be performed to clarify the doses that would be necessary and the proper way of delivery that could offer an efficient bioavailability and long-term tolerability of these FA.

## Figures and Tables

**Table 1:** Properties of ω3 fatty acids in the therapeutic approach of inflammatory bowel disease.

Reference	Model	Investigation or Intervention	Major Findings	Conclusions
Diab et al. 2019 [4]	Controlled clinical trial. Colon biopsies were taken from 15 treatment-naïve UC patients (6 women, 9 men; 14–69 years), five deep remission UC patients (5 men; 41–70 years), and 10 healthy controls (4 women, 6 men; 25–86 years).	Thirty-five oxylipins and 11 eCBs were quantified. A reverse transcription-polymerase chain reaction measured levels of mRNA for ten cytokines.	Levels of ω6-related oxylipins were significantly elevated in treatment-naïve patients compared with controls, whereas the levels of ω3 eCBs were lower. 15S-HETrE was significantly upregulated in UC deep remission patients. All investigated cytokines had significantly higher mRNA levels in the inflamed mucosa of treatment-naïve UC patients. Cytokine gene expression was positively correlated with several ω6 AA-related oxylipins, whereas a negative correlation was found with lipoxin, prostacyclin, and the eCBs.	Increased levels of ω6-related oxylipins and decreased levels of ω3-related eCBs are associated with the debut of UC. This highlights the altered balance between pro- and anti-inflammatory lipid mediators in IBD and suggests potential targets for intervention.
Scaioli et al. 2018 [17]	Randomized clinical trial with sixty UC patients (29 men and 31 women, 18 years and older), with a partial Mayo score < 2 and FC ≥ 150 μg/g, in stable therapy for at least three months.	Patients were randomly divided into groups with EPA-FFA (500 mg, twice/d) or placebo for six months. Evaluation with colonoscopy was taken at baseline. Assessments of fecal calprotectin and clinical parameters were performed at baseline of the study, after 3 months, and after 6 months. Relapse UC patients underwent another colonoscopy.	Calprotectin showed a reduction of 100 points at six months from the baseline (63.3% in the EPA-FFA group and 13.3% in the placebo group). It was observed the maintenance of clinical remission at six months (76.7% in the EPA-FFA group and 50% in the placebo group). No serious adverse events were observed.	The use of EPA-FFA was essential to reduce FC and no serious adverse events were observed. This agent can be used to induce and maintain symptom-free remission in UC patients.
Prossomariti et al., 2017 [18]	Pilot Study with twenty UC patients (13 men and 7 women, 18–70 years), in stable clinical remission and with FC > 150 µg/g.	Supplementation with EPA-FFA 2 g/daily for 90 days.	EPA-FFA intervention decreased the levels of FC at 90 days. Individuals with FC > 150 µg/g (at 90 days) were classified as nonresponders. EPA-FFA ameliorated endoscopic and histological inflammation and induced expression of IL-10, HES1, SOCS3, and KLF4 in compatible patients. EPA-FFA partially redressed long-term UC-driven microbiota composition.	The supplementation with EPA-FFA decreased mucosal inflammation, induced differentiation of goblet cells, and modulated the composition of the intestinal microbiota in patients with long-standing UC.
Yasueda et al. 2016 [19]	Open-label clinical trial with five CD patients in remission (4 men and one woman; 29–60 years).	Intake of 100 mL of ω3 emulsifying formulation test (IMARK S^®^) daily/28 d. After a month washout period, patients drunk two bottles of the formulation/daily/28 days. Anthropometric and biochemical evaluations were performed before and after each intervention.	The formulation was safe with minimal side effects. Bodyweight and body mass index were not modified. CD activity index scores decreased after ingestion of one bottle of the formulation. Blood evaluations did not show severe side effects.	Supplementation with the formulation test can be safe and useful for maintaining remission in CD patients.
Wiese et al. 2016 [20]	Controlled clinical trial with 101 UC subjects (45 men and 56 women, 27–58 years) and 23 controls (9 men and 14 women, 37–65 years).	Dietetic inquiries, serum, and colonic tissue samples were collected. Histologic injury and the activity index (Mayo Disease) were assessed. Cytokines from serum and tissue were measured. Serum FA were evaluated.	UC individuals showed increased total OA and decreased AA intake when compared with the control group. Reduced percentages of SFA and AA, and higher MUFA, OA, EPA, and DPA were also observed. The levels of cytokine in the tissue were directly associated with SFA and inversely correlated with PUFA, EPA, and DPA in UC subjects, but not with controls. 5-aminosalicylic acid therapy blunted these associations.	There were differences in serum FA in individuals with UC that correlated with proinflammatory tissue cytokines suggesting that fatty acids can affect the production of cytokines and therefore be immunomodulators in UC.
Scaioli, et al. 2015 [21]	Controlled clinical rial with ten UC (4 men and 6 women, 24–44 years) and 10 CD individuals (6 men and 4 women, 28–50 years) and 15 HV (5 male and 10 female, 20–36 years).	Patients received 2 g/d of EPA-FFA for eight weeks.	There was rapid incorporation of EPA into plasma phospholipids by two weeks and high incorporation into membranes of RBC (4% total FA content). There was a concomitant reduction in relative ω6 PUFA content. The elongation and desaturation of EPA (into DHA) was apparent. DHA content also augmented in the membranes. EPA-FFA was well tolerated, and no significant differences in the pharmacokinetic profile of ω3 PUFA incorporation were found between IBD patients and HV.	EPA can be considered the “universal donor” concerning key ω3 PUFAs and the enteric-coated formulation test allows long term treatment with a good level of compliance.
Chan et al. 2014 [22]	Multi-center prospective cohort study with 229,702 healthy participants (20–74 years) from nine European centers between 1991–1998 and monitored until 2004 (data on gender were not precise). None of the controls possessed UC, microscopic or uncertain colitis.	Dietary intake of DHA was measured at baseline (validated food frequency questionnaires). The population of the study was monitored to identify participants who developed CD.	Seventy-three individuals developed CD (64% woman), which is equivalent to 4 new cases/100,000 inhabitants/year. The consumption of ω3 may influence the development of CD	The intake of DHA was statistically significant inverse related to the development of CD.
Ananthakrishnan et al. 2014 [23]	Prospective Cohort study with 170,805 women over 26 years and 3,317,338 person-years of follow-up.	Diet was prospectively evaluated using a validated semi-quantitative food frequency questionnaire. Self-reporting of UC or CD was confirmed through medical report review.	Among the women sample, 338 incident cases of UC (10 new case/100,000/year) and 269 incident cases of CD (8/100,000/year). Intake of ω6 and ω3 was not associated with the risk of developing CD or UC. On the other hand, high long-term intake of trans-unsaturated FA was related to an augmented incidence of UC.	Saturated or unsaturated fat, total fat, or individual PUFA intake does not influence the risk of CD. Trans-unsaturated FA is correlated with UC.
Costea et al. 2014 [24]	Randomized controlled trial with 182 children recently diagnosed with CD (111 men and 71 women, 10–16 years), diagnosed before age 19, and 250 controls (Caucasians) (122 men and 128 women, 10–16 years).	The children’s responses to the questionnaire on daily consumption were evaluated. The raw intakes of EPA, DPA, and DHA and AA (ω6) were adjusted for energy intake. The ratio of AA/(EPA, DPA, DHA) was calculated.	Children who intake higher dietary ratio of ω6/ω3 were vulnerable for developing CD if they also presented specific variants of CYP4F3 and FADS2 genes. The findings implicate diet–gene interactions in the pathogenesis of CD.	These findings suggest that preventive dietary intervention could be targeted to specific subgroups (based on PUFA metabolic genes) of IBD.
Pearl et al., 2014 [25]	69 patients with active UC, (35 men and 34 women, 44–47 years), 16 with UC in remission (8 men and 8 women, 43–50 years) and 69 control subjects matched by age and gender (35 men and 34 women 45–48 years). Age 16–80 years. Exclusion criteria: age less than 16 or greater than 80 years.	Biopsies from colonic mucosa were obtained from UC patients and controls. Inflammation was analyzed endoscopically and histologically. FA (esterified and non-esterified) were evaluated.	Inflamed mucosa showed higher AA (*p* < 0.001), lower EPA (*p* < 0.010); higher DPA and DHA, and lower LA and α-LNA contents (all *p* < 0.001), compared to controls. There was a significant association between the severity of inflammation and contents of AA, DPA, and DHA (positive correlations), and LA, α-LNA, and EPA (negative correlations).	Increased AA, AA: EPA ratio, DPA and DHA and lower LA, α-LNA, and EPA were observed in inflamed mucosa and correlate with severity of inflammation, suggesting a modification in FA metabolism. This finding may offer a novel target for intervention.
Bassaganya-Riera et al., 2012 [26]	Double-blind, placebo-controlled, randomized trial with thirteen CD patients (2 men and 11 women) with mild to moderately active pattern. Age 25–61 years.	Patients used CLA 6 g/day/12 weeks. Mononuclear cells and cytokines were analyzed at baseline, six, and twelve weeks after initiation of the treatment; CDAI and IBDQ were also performed.	The use of CLA significantly reduced the production of TNF-α, IFN-γ, IL-17, and lymphoproliferation at week 12. There was a significant reduction in CDAI and increase in IBDQ on week 12.	The oral use of CLA was well-tolerated and reduced the capacity of peripheral blood T cells to release pro-inflammatory cytokines, increased quality of life, and reduced disease activity, index.
Grogan et al. 2012 [27]	Double-blind randomized controlled trial with 34 children (20 boys and 14 girls) newly diagnosed CD Age 5–16 years.	Children were randomized to the elemental formula (EF), where the diet came from amino acid sources and had a higher concentration of LA, or polymeric formulation (PF), where the dietary source was whole protein and had higher concentration of ALA/six weeks. Change in the PCDAI, FC, and plasma FA were measured at 0 and six weeks. Patients were followed up for two years. Time and treatment choice for the first relapse were documented.	Ninety-three percent of the patients achieved remission in the EF group and 79% in the PF group. One-third of the patients maintained remission for two years. With PF, an increase of EPA and ALA was found with a reciprocal decrease in AA. EF, EPA, and AA levels decreased with a significant reduction in DHA. FC decreased significantly but did not normalize at the end of week 6.	Results did not present a significant difference between the use of EF or PF in promoting remission. Changes in plasma PUFA status were subtle and relevant.
Wiese et al., 2011 [28]	Randomized controlled trial with 20 patients with active CD and stable medication. Eligible patients were 18 years or older (4 men and 16 women).	Patients received 16 oz of IBDNF/day/four months.	The results showed that a dietary supplement enriched with fish oil, prebiotics, and antioxidants in CD resulted in increased fat-free and fat mass deposition, improved vitamin D status, and led to an improvement in quality of life and lower disease activity.	IBDNF exhibits potential to be used as an adjuvant in the treatment of CD patients.
Grimstad et al., 2011 [29]	Open study, nonrandomized, clinical trial with 12 UC patients (5 men and 7 women, 35– 65 years.	600 g of salmon was consumed weekly/8 weeks) and a dietary questionnaire, SCCAI, sigmoidoscopy evaluation, FC serum inflammatory markers, and rectal biopsy and plasma FA profiles were performed before and after the dietary intervention.	The levels of C20:4 ω-6 AA in biopsies after intervention were associated with endoscopy and histology and scores. The levels of ω3 PUFAs, C20:5 ω3EPA, C22:6 ω3DHA, and the ω3/ω6 ratio increased in plasma and rectal biopsies. The AIFAI increased both in plasma and biopsies accompanied by a significant reduction in SCCAI.	The intake of salmon reduced SCCAI and AIFAI and showed a tendency to decrease the levels of CRP and homocysteine. For these reasons, it may have beneficial effects on disease activity in patients with mild UC.
Uchiyama et al., 2010 [30]	Controlled clinical trial with 20 initial-onset IBD patients (12 UC patients (3 men, 9 women, mean age of 32.9 years) and 8 CD patients (5 men, 3 women, mean age: 29.0 years) who had not undergone any dietary intervention, and after 230 patients (168 UC patients (90 men, 78 women, mean age: 36.0 years) and 62 CD patients (46 men, 16 women, mean age: 34.6 years) in remission for 12 months	The intake of FA in the erythrocyte membrane was evaluated before and after the intervention with diet therapy involving the use of a ω3DP. This regimen, to achieve a ω3/ω6 ratio of 1 V6 PUFA intake was restricted to 50% of the mean intake, and ω3 PUFA intake was increased 2-fold in comparison with the mean, after which the activity of the disease was evaluated after 12–18 months in remission patients.	In the 20 initial-onset patients, the ω-3/ω-6 ratio significantly augmented after the intervention. The ratio in the remission group (*n* = 145) in the follow-up group was significantly higher than in the relapse group (*n* = 85). The ratio was significantly decreased in patients who suffered a relapse after the beginning of the treatment.	ω3DP significantly decreased the erythrocyte membrane ω3/ω6 ratio in IBD individuals and was significantly higher in the remission group. These findings suggest that ω3DP alters the FA composition of the cell membrane and influences clinical activity in IBD patients.

UC: ulcerative colitis; eCBs: endocannabinoids; 15S-HETrE: 15S-hydroxy-eicosatrienoic acid; EPA-FFA: eicosapentaenoic acid as free fatty acid; FC: fecal calprotectin; IL-10: interleukin-10; HES1: transcription factor HES1; SOCCS3: suppressor of cytokine signaling 3; KLF4: Kruppel-like factor 4; CD: Crohn’s disease; FA: fatty acid; SFA: saturated fatty acid; MUFA: monounsaturated; AA: arachidonic acid; OA: oleic acid; DPA: docosapentaenoic acid; HV: healthy volunteers; RBC: red blood cell; IBD: inflammatory bowel disease; NOS: FA-enriched; LTB5: leukotriene B5; LTB4: leukotriene B4; CYP4F3, FADS1 and FADS2: variants of genes that control polyunsaturated fatty acid metabolism; LA: linoleic acid; α-LNA: α-D-configured locked nucleic acid; CON: control group; ICU: intensive care unit; CLA: conjugated linoleic acid; CDAI: CD activity index; IBDQ: inflammatory bowel disease questionnaire; PCDAI: pediatric Crohn’s disease activity index; ALA: α-linolenic acid; DHA: docosahexaenoic acid; EPA: eicosapentaenoic acid; PUFA: Polyunsaturated fatty acids; IBDNF: inflammatory bowel disease nutrition formula; SCCAI: simple clinical colitis activity index; AIFAI: anti-inflammatory fatty acid index; CRP: C-reactive protein; ω-3DP: ω3PUFA food exchange table.
